# Histone Post-Translational Modifications and DNA Double-Strand Break Repair in Neurodegenerative Diseases: An Epigenetic Perspective

**DOI:** 10.3390/biology14111556

**Published:** 2025-11-06

**Authors:** Arefa Yeasmin, Mariana P. Torrente

**Affiliations:** 1 Department of Chemistry & Biochemistry, Brooklyn College, Brooklyn, NY 11210, USA; 2 PhD Program in Biology, The CUNY Graduate Center, New York, NY 10016, USA; 3 PhD Programs in Biology, Biochemistry & Chemistry, The CUNY Graduate Center, New York, NY 10016, USA

**Keywords:** central nervous system, epigenetics, histone post-translational modifications, DNA damage response, DNA repair, homologous recombination, non-homologous end joining, neurodegenerative diseases, ALS/FTD, AD, PD, MS, HD

## Abstract

DNA damage is a hallmark of the fatal process of neurodegeneration in the central nervous system (CNS). As neurons are terminally differentiated, they accumulate metabolic and oxidative burdens over their whole life span. Unrepaired DNA develops into DNA double-strand breaks (DSBs), which are repaired through homologous recombination (HR) or non-homologous end joining (NHEJ). Being post-mitotic and unable to normally undergo HR, damage and defective repair is especially burdensome to CNS neurons. Current research has not produced treatment to prevent and halt progression of neurodegeneration. Hence, novel targeting strategies are desperately needed. Recent investigations in histone post-translational modifications (PTMs) reveal new mechanistic insight and highlight unexplored targets to ameliorate neurodegeneration. As various histone PTMs dictate and facilitate DSB repair, they represent an underexploited area in investigating DNA damage and incorrect repair aiding neurodegeneration. Here, we review the histone PTM alterations in several neurodegenerative diseases: Amyotrophic Lateral Sclerosis/Frontotemporal Dementia, Parkinson’s Disease, Alzheimer’s Diseases, Multiple Sclerosis, and Huntington’s Disease. These findings emphasize that histone PTM alterations can enable an aberrant DNA damage response (DDR) leading to neurodegeneration. Further research into the connections between histone PTMs and DNA damage in decaying neurons will illuminate novel targets to dampen the aberrant DDR and promote neuronal survival.

## Simple Summary

Neurodegeneration is a fatal process often involving damage to the genome. The post-mitotic status of neurons make DNA repair an essential and crucial process to prevent neurodegeneration. Histone post-translational modification is an epigenetic mechanism that aids in DNA repair, dysregulation of which can contribute to persistent DNA damage followed by neuronal death. This review summarizes histone post-translational modifications involved specifically in DNA double-strand break repair and alterations in the level of certain DNA repair-related histone marks in various neurodegenerative diseases. Further evaluation of histone modifications associated with DNA repair in relevant disease models would provide mechanistic insights into neurodegeneration, as well as reveal novel targets and preventative strategies.

## 1. Introduction

Neurodegeneration is a heterogeneous process commonly influenced by aging, environmental exposures, and genetic mutations, culminating in the death of neurons [1]. Common molecular etiologies include protein misfolding and aggregation, dysregulation of intracellular protein localization, aberrant protein signaling, and damage to genomic DNA, arising from external or internal offenses [2,3,4,5,6,7,8]. Various protective processes, such as the unfolded protein response (UPR), autophagy, and DNA repair mechanisms, are in place to maintain cellular homeostasis [3,9,10]. The impairment of these processes contributes to disease pathologies by exacerbating the damage [10,11,12]. Current therapies are able to relieve symptoms but fail to stop disease progression [13,14,15]. As such, there is an urgent need for novel therapeutic avenues. Investigating novel aspects of disease mechanism would enhance the search for targetable pathways and molecular players.

The central nervous system (CNS), comprising the brain and the spinal cord, bears an extra burden in instances of DNA damage, as many of the CNS cell populations are at their terminal cell fate [12,16,17]. Cells in the CNS rarely undergo mitosis, usually limited to progenitor cells and occasional, extraneous and/or erroneous cases, such as neuronal mitotic re-entry [18,19]. Because of their high metabolic demands as well as genomic activity throughout their long lifespan, neurons accumulate DNA damage [20,21]. Reactive oxygen species, radiation, environmental toxins, and normal molecular processes such as transcription can also result in DNA damage [11,21]. Impairment of DNA repair mechanisms increases the probability for neurons to accumulate DNA double-strand breaks (DSBs). Impairment of DSB repair often leads to cell death. Indeed, DNA damage and aberrant repair is involved in various neurodegenerative diseases, which raises an argument for re-examination of DNA damage and repair in neurodegeneration [12,17,20].

Epigenetics is the study of heritable changes in gene expression occurring in an organism without any changes to the genome [22,23,24]. Eukaryotic DNA is precious code that requires careful packaging and protection to enable proper cellular and overall organismal function. The histone octamer, consisting of two H2A and H2B dimers and an H3 and an H4 tetramer, helps to package and maintain DNA. DNA wraps around histones and forms the nucleosome [25]. Further packaging of the DNA–histone complex results in chromatin and, eventually, chromosomes [26]. The DNA–histone interaction plays a crucial role in determining the compaction level of chromatin. The post-translational modification (PTM) of histones is a prominent form of epigenetic regulation that modulates the DNA–histone interaction to aid and guide virtually all cellular processes, including DNA replication, transcription, and DNA repair [26,27,28].

The N-terminal tails of histones commonly undergo various chemical modifications, such as methylation, phosphorylation, and acetylation, among others [26,28]. Acetylation neutralizes the positively charged lysine residues on histones attracted to the negatively charged DNA, thereby reducing the strength of interaction between DNA and histones and making DNA more accessible. Methylation, which can be installed up to three times on the same lysine or arginine residue, provides steric hindrance, preventing proteins from binding to the DNA, depending on the position where it is applied. Furthermore, histone methylation often acts as a binding platform for other proteins. Phosphorylation contributes a negative charge to serine, threonine, and tyrosine residues, reducing binding strength between DNA and histones [29]. These PTMs often coexist on the same histone tail at different residues, culminating in a code that speaks to specific genomic processes [30]. Adding another layer of complexity to this code, there are histone-modifying enzymes (HMEs) that read, write, and erase these modifications to elicit certain chromatin conformations, DNA accessibility, and signaling cascades to facilitate cellular processes [31,32]. For example, tri-methylation of histone H3 at lysine 4 (H3K4me3), installed by lysine methyltransferases (KMTs) such as components of the SET1/MLL complex and recognized by chromodomain proteins such as CHD1, is linked to active gene expression, as it is found at the promoters of active genes [33,34,35,36,37]. Histone acetyltransferases (HATs) such as the components of the SAGA complex install H3K9ac at active gene expression sites as well, and this is often recognized by bromodomains such as BRD7 [38,39]. Gene repression, on the other hand, has been linked to marks such as H3K9me3 and H3K27me3. An interplay between the writers and erasers, as well as the readers, enables the dynamic regulation of chromatin that allows DNA to seamlessly dictate cellular functions in a localized and time appropriate manner [30,40].

DNA damage and repair uses the language of histone PTMs to monitor and maintain the genomic code [27,41,42,43,44]. Histone PTMs can act as a signaling mechanism that aids in sensing DNA damage, choosing the repair pathway, and initiating repair [41,43,44]. They can also serve as scaffolds to recruit repair proteins and facilitate protein–DNA interactions to promote repair processes [41,45,46]. Over the years, a handful of histone PTMs have been investigated for their role in DNA damage response and repair. For instance, phosphorylation of the H2AX histone variant γ-H2AX is a well-known marker of DNA DSBs that initiates the DNA repair cascade and enables the accumulation of repair proteins at these sites. Depending on the type of damage and the cell’s mitotic status, these PTMs and their levels can vary, especially in diseased contexts. Aberrant histone PTMs and PTM levels, therefore, can impair DNA repair and possibly lead to cell death by furthering damage persistence. In the specific case of DSBs, the deleterious nature of this type of damage, combined with the lack of mitotic potential of neurons, renders the repair dysregulation severely damaging in the CNS.

Here, we review the different DNA damage repair pathways and histone PTMs engaged in the CNS with a focus on DSBs in various neurodegenerative diseases. DSBs are the most severe type of genomic damage that often leads to mutations such as deletions, insertions, and translocations, as well as cell death if left unrepaired [12,47]. For histone PTM dysregulation exploration in single-strand breaks and oxidative damage, we direct the reader to several excellent reviews on the topic [48,49,50,51]. Neurons are equipped to handle single-strand breaks in DNA. However, as their lifelong function results in accumulation of damage from oxidative causes and high metabolism, unrepaired single-strand breaks and overwhelming oxidative damage both cause accumulation of DNA DSBs, which lead to cell death. Therefore, it is imperative to re-examine the dysregulation of DSB repair in neurons to ascertain its role in neurodegeneration. Histone PTMs present a novel lens to approach this phenomenon based on the different cascades that these marks direct and support [52]. We highlight how certain histone PTMs and their dysregulation play a key role in specific DNA repair pathways involved in neurodegeneration. The evidence reviewed underscores that histone PTM alterations contribute to an aberrant DNA damage response (DDR) leading to neurodegeneration. Deepening our understanding of the relationships between histone PTMs and DNA damage in the context of neurodegeneration can reveal novel openings to restore normal DDR and enhance neuronal survival.

## 2. DNA Double-Strand Break Repair Pathways: Homologous Recombination and Non-Homologous End Joining

DNA damage is an early sign of neurodegeneration in Alzheimer’s, Parkinson’s, ALS, and Huntington’s diseases, as well as Amyotrophic Lateral Sclerosis (ALS), among others [4,6,8,53]. Damage can include base alterations, base mismatching, thymine dimerization, single-strand breaks, and double-strand breaks, among others [11]. On the other hand, repair generally involves damage recognition, repair initiation, and repair including DNA synthesis, followed by ligation [4,54].

**Figure 1:**
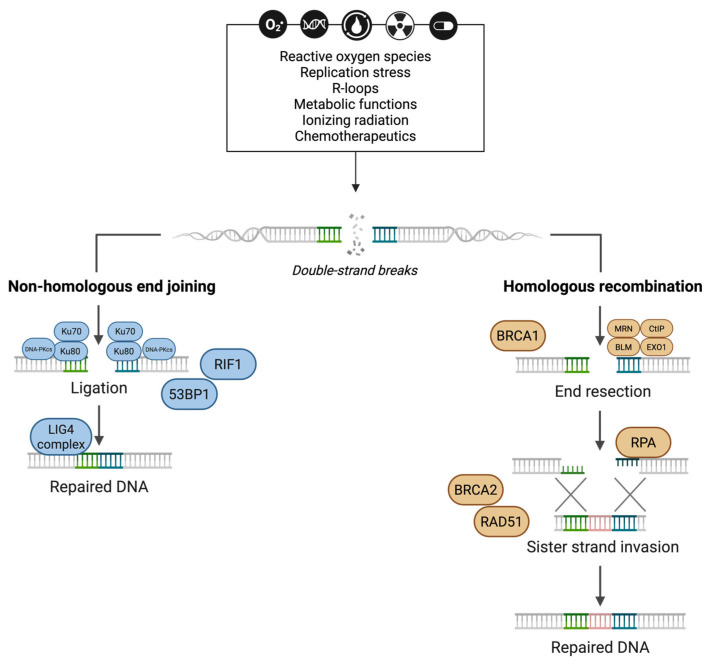
**DNA double-strand break repair pathways.** Double-strand breaks arising from unrepaired single-strand breaks or ionizing radiation and chemotherapeutics are repaired through either homologous recombination (HR) or non-homologous end joining (NHEJ) through a cell-cycle-dependent manner. Figure created in BioRender.

Double-strand breaks (DSBs) are the most severe form of DNA damage, especially in the CNS [55]. Interestingly, while generally negative, DSBs can also aid in processes such as class switch recombination and memory formation in the CNS [56]. DSBs can result from damage-inducing agents or from persistent unrepaired single-strand breaks. For instance, R-loops—DNA–RNA hybrids formed during transcription—often lead to DNA damage by exposing single-stranded DNA to endonucleases and can be detrimental to repair machinery, as R-loops themselves limit strand access/dynamics [57]. Cells repair DSBs through two main pathways: homologous recombination (HR) and non-homologous end joining (NHEJ) (Figure 1). There are variations to these pathways based on context. While HR is the high-fidelity repair pathway ensuring error-free repair, it is only limited to late S and G2 phases of the cell cycle, as sister chromatids are necessary as templates for this repair pathway [58,59]. On the other hand, while NHEJ is error-prone and can lead to deletions and additions alike, it can be engaged in all phases of the cell cycle. Correct repair pathway choice and subsequent faithful repair are crucial in neurons, as these cells are terminally differentiated and normally limited to the more error-prone NHEJ pathway.

DSBs are most often addressed by NHEJ. NHEJ begins with the Ku70/Ku80 heterodimer binding at DSB sites [11]. Subsequent recruitment of 53BP1 and RIF1 solidifies the NHEJ pathway choice and prevents end resection. XRCC4 then stabilizes the broken strand for repair. The XRCC4-Artemis-DNA PKcs-DNA ligase 4 (LIG4) complex then ligates the strands completing repair [58,60]. During the DNA ligation in NHEJ, errors can occur via translocations, expansions, and deletions, rendering NHEJ highly error prone.

BRCA1 is also simultaneously recruited with 53BP1 at DSB sites in an asymmetric fashion [61]. BRCA1 inhibits 53BP1 and promotes HR. HR begins with the accumulation of the MRN complex (MRE11/RAD50/NBS1) [11]. Together with BRCA1, the MRN complex, CtIP, EXO1, and BLM helicase resect DNA and generate single-stranded 3′ DNA overhangs [62]. Furthermore, replication protein A (RPA) accumulates quickly at the break site and coats the overhang to prevent DNA folding and digestion to promote RAD51 recombinase (RAD51) accumulation [16]. In repair during replication, ATR binds to RPA at the ssDNA to engage ATR-Chk1 to induce cell cycle arrest. BRCA2 aids in RAD51 accumulation, which subsequently enables sister strand invasion and homology-based repair. The use of a sister chromatid as a template in HR promotes a more faithful DNA repair.

## 3. DNA Damage in Neurodegenerative Diseases

DNA damage is a hallmark of neurodegeneration, commonly observed as both a cause and promoter of neuronal death. In many cases, DNA damage initiates molecular cascades involved in various neurodegenerative diseases. Below, we showcase evidence for neurodegenerative diseases’ association to DNA damage.

### 3.1. Amyotrophic Lateral Sclerosis/Frontotemporal Dementia

Amyotrophic Lateral Sclerosis and Frontotemporal Dementia (ALS/FTD) are fatal neurodegenerative diseases sharing various pathological characteristics ranging from genetic mutations to molecular phenotypes that comprise and make up a disease continuum [63]. ALS affects upper motor neurons in the motor cortex and lower motor neurons in the brainstem motor nuclei and spinal cord anterior horn, resulting in muscular failures and atrophy, loss of motor coordination, and respiratory failure. FTD, on the other hand, affects the frontal and temporal lobe responsible for cognitive processes and memory formation, resulting in language impairments and behavioral changes. While most cases occur sporadically, there are many genes associated with ALS/FTD, including *SOD1* (superoxide dismutase 1), *FUS* (fused in sarcoma), *TAU* (tubulin associated unit), *TDP-43* (TAR-DNA-binding protein 43), and *C9orf72* (Chromosome 9 open reading frame 72) [64,65,66,67,68,69,70,71]. Cellular etiologies associated with mutations in these genes range from ER stress, mitochondrial dysfunction, and disrupted RNA metabolism to protein aggregation in the cytoplasm [72,73,74,75]. Notably, as many ALS/FTD genes participate in addressing DNA damage repair, DNA damage and defective repair are hallmarks of ALS/FTD.

#### 3.1.1. SOD1

Superoxide dismutase 1 (SOD1) is one of the three superoxide dismutases responsible for removing reactive oxygen species produced from general metabolic functions in a cell. SOD1 is primarily localized in the cytoplasm, but localizes to the nucleus when a high oxidative environment occurs there [70]. Mutations in SOD1 and loss of its antioxidant properties could lead to persistent reactive oxygen species (ROS)-mediated DNA damage and overwhelm DNA repair pathways [76]. Furthermore, mutant SOD1 presence has also been linked to transport deficiencies of DNA repair proteins, such as p53, HDAC1, and even FUS, furthering SOD1’s link to DNA damage [77]. Indeed, ALS patient spinal cord, frontal cortex, and striatum, as well as a transgenic mousemodel expressing SOD1-G39A, show increased levels of 8-hydroxy-2′deoxyguanosine (8OH2′dG), a marker of oxidative DNA damage [78,79]. Moreover, overexpression of mutant SOD1 in motor neuron-like NSC34 cells represses SpeedyA1—a cell death suppressor—in response to DNA damage, rendering the cell more susceptible to persistent DNA damage and death [80]. Altogether, these findings suggest that DNA damage plays an active role in SOD1-ALS pathology.

#### 3.1.2. FUS

Fused in sarcoma (FUS) is an RNA-binding protein, mostly localized to the nucleus, known to be involved in transcription and controlling gene expression of spliced products of genes [81,82]. FUS has also been implicated in mRNA transport out of the nucleus, as well as DNA repair [82]. Its RGG-rich composition makes it likely to aggregate and promotes its binding to other aggregation-prone proteins, such as the RNA-binding protein TDP-43, another ALS-associated protein [64]. FUS plays a role in DNA repair by being recruited to nuclear DNA damage sites following its phosphorylation by DNA-dependent protein kinase (DNA-PK). In ALS, pathogenic mutations cause FUS to aberrantly aggregate in the cytoplasm, leading to loss of nuclear FUS function and impaired DNA repair capacity [7,83]. More importantly, FUS participates in DNA repair via its interaction with HDAC1 and by getting phosphorylated in response to DSB formation [83,84]. Owing to its function in DNA repair, mutant FUS results in repair defects such as ligation failure in strand repair [85]. In induced pluripotent stem cell (iPSC)-derived motor neurons, FUS aggregation is preceded by PARP-mediated DNA signaling failure [86,87]. Thus, targeting the DDR signaling may be beneficial in tackling FUS-ALS neurodegeneration. Interestingly, the disruption of RNA processing and DDR together exacerbate the susceptibility of neuronal death in FUS pathology [82]. Various FUS models show increased DSBs as further evidence for making a strong case for DNA damage contributing to and furthering neurodegeneration in FUS-ALS/FTD [81,84,86,88].

#### 3.1.3. TDP-43

Transactive response DNA-binding protein 43 (TDP-43) is a DNA/RNA-binding protein involved in numerous genomic processes ranging from RNA metabolism, gene expression, and R-loop clearance to DNA repair [89,90,91]. Although mutations in the *TARDBP* gene account for a subset of ALS cases, the majority of ALS cases tend to show TDP-43 pathology as well [90]. TDP-43 functions in DNA repair, including R-loop resolution as well as facilitation of NHEJ [91,92]. TDP-43-ALS/FTD pathology usually involves the cytoplasmic aggregation of mutant TDP-43 and nuclear depletion of wild type TDP-43. Mislocalization and aggregation of TDP-43, including sequestration of other essential proteins in the cytoplasm, exacerbates the loss of these protective measures against DNA damage. Spinal cord tissue from a patient with a Q331K mutation, as well as SH-SY5Y neuroblastoma cells with the same mutation, displayed elevated γ-H2AX levels and increased activation of DNA repair proteins [93]. ALS patient-derived fibroblasts and cortical neurons from a TDP-43 ALS/FTD murine model both show decreased NHEJ repair, indicating not only persistent DNA damage but also defective repair [92,94].

#### 3.1.4. C9ORF72

The G_4_C_2_ hexanucleotide repeat expansion in the Chromosome 9 open reading frame 72 (*C9orf72*) is the most frequent mutation in ALS/FTD [95]. C9ORF72 participates in autophagy, inter/intracellular vesicle trafficking, and actin dynamics [63]. Bidirectional transcription of the expansions results in long sense and antisense RNA strands that tend to aggregate into RNA foci. The expanded RNA transcripts then undergo repeat associated non-AUG (RAN) translation, resulting in five dipeptide repeat proteins (DPRs). Thus, C9 loss of function often synergizes with gain-of-function toxicity pathways arising from cytoplasmic inclusions of both RNA and DPRs, culminating in neuronal death [65,96,97,98]. Interestingly, C9ORF72 itself has been linked to having DNA repair functions via autophagy activity towards TDP-43. C9 loss of function, therefore, allows for accumulation of DNA damage [99]. Gain-of-function mechanisms, however, appear to be more important in DNA damage-related pathology in C9-ALS/FTD. C9-ALS patient motor neurons and neuronal cells expressing DPRs display elevated levels of DSBs, as well as repair proteins [100]. Furthermore, the expanded RNAs sequester various repair-associated proteins while the RAN DPRs form toxic cytoplasmic inclusions which exacerbate the DNA damage arising from the expansion [101,102]. DPRs can disrupt nucleocytoplasmic transport, thereby potentially affecting recruitment of repair proteins to the nucleus in response to DNA damage [98,103]. Increased DNA damage is also seen in iPSC-derived motor neurons ectopically treated with DPRs, as well as fly neurons expressing DPRs [104]. Single-cell whole genome sequencing in the prefrontal and premotor cortex of C9-ALS and C9-FTD patients show increased oxidative-damage-based somatic single nucleotide variant (sSNV) mutations and single-strand break based somatic insertion//deletion (sInDel) mutations [105]. All this evidence indicates that DNA damage and a dysregulated DDR are important in C9-ALS/FTD pathology.

### 3.2. Parkinson’s Disease

Parkinson’s disease (PD) is most often characterized by the impairment of motor functions due to loss of dopaminergic neurons [5,53]. Clinical manifestations include motor symptoms such as resting tremor, muscle stiffness, and gait and posture abnormalities, as well non-motor symptoms such as depression, autonomic dysfunction, and loss of smell, among others [53]. The demise of dopaminergic neurons often arises from accumulation of Lewy bodies consisting of misfolded and aggregated α-Synuclein (α-Syn) protein most often linked to mutations in the *SNCA* gene. PD is also linked to the loss of ubiquitin ligase function of PRKN, the protein product of Parkin, that results in defective mitophagy [106]. PD can be caused by oxidative stress, environmental toxins, and a-Syn aggregation, among others [107,108,109]. Due to the role of these proteins in DNA repair processes, loss of function of PRKN, a-Syn, and DJ-1 (Parkinsonism associated protein deglycase) has been linked to DNA damage in PD [59,110,111]. Indeed, knocking out *SNCA* in a mouse PD model rendered DSB repair defective despite damage foci formation [110]. Two different in vivo mouse PD models display increased 53BP1 levels, which plays important roles in facilitating double-strand break repair, indicating engagement of DNA DSB repair [60,61,107,112]. Therapeutics against DNA-damage-mediated neurodegeneration in PD have been attempted by chemically inhibiting ATM, an important kinase in the repair cascade responsible for phosphorylating H2AX and repair proteins such as 53BP1 [112,113,114]. While current evidence is still limited, it suggests a role for DNA damage in PD neurodegeneration.

### 3.3. Alzheimer’s Disease

Alzheimer’s disease (AD) is the most common form of dementia. Clinical symptoms in AD involve gradual cognitive decline, including memory loss, disorientation, mood swings, and language impairments, among others. AD is characterized by intracellular fibrillary tangles and extracellular plaque deposits [115]. The plaque deposits are made up of misfolded Amyloid-β (Aβ) proteins, while the fibrillary tangles are often comprised of mutant, hyperphosphorylated TAU. Mutations in *APP*, *PSEN1*, and *PSEN2*, among others, are thought to promote Aβ plaque formation [116]. Moreover, the Aβ plaque formation triggers TAU fibrillation, resulting in a combined pathology in AD [117,118]. The current literature also implicates DNA damage in various AD contexts.

#### 3.3.1. Amyloid-β

Amyloid-β is produced as a metabolic byproduct of the cleavage of amyloid precursor protein and is normally cleared by cells [119]. In the event of defective clearance, Aβ accumulates in the extracellular cortex space and affects neurites and astrocytes [119]. As cellular transport at neurites and astrocytic neurotransmitter recycling processes support neuronal function and health, Aβ plaque formation leads to neurodegeneration by impeding these processes [120]. Transgenic mice expressing mutant human APP (hAPP), AD patient hippocampi, and neurons from post-mortem AD brain display increased DSBs [8,121,122]. Furthermore, AD patient brains and mice expressing hAPP show decreased levels of BRCA1, a key repair protein involved in homologous recombination [123]. Moreover, primary neurons treated with Aβ display 50% reduction in BRCA1 levels, indicating potential defective HR [124]. Notably, oxidative damage is prominent in AD cases and often results in ssDNA breaks [125]. As NHEJ is less effective due to Aβ pathology via DNA-PK deficiency, the unrepaired ssDNA breaks can accumulate into DSBs, showing that both increased DNA damage and defective repair playing a role in Aβ-AD neurodegeneration [125].

#### 3.3.2. TAU

Tubulin-associated unit (TAU) is one of the first microtubule-associated proteins to have been discovered [126]. Mutations in TAU renders the cell deficient in intracellular transport, among other cytoskeletal processes [126]. As many crucial processes require translocation of proteins across the cell, TAU mutations have a significant effect on neuronal health. For example, TAU mutations can hinder movement of repair proteins from the cytoplasm to the nucleus in cases of DNA damage [127]. Specifically, hyperphosphorylated TAU dissociates from microtubules and misfolds and mislocalizes to the nucleus, rendering tubulins destabilized [128]. Furthermore, mutant TAU can cause defective DNA repair and cytoplasmic DNA accumulation, which then prevents proper TAU association with microtubules, indicating a potential feedback loop of TAU in DNA damage contexts [129,130]. As AD patient cortices with hyperphosphorylated TAU also show increased DSBs and depletion of endogenous TAU results in increased DSBs, TAU is thought to play a role in DNA maintenance and repair, as well among other nuclear roles [126,127,131].

### 3.4. Multiple Sclerosis

Multiple Sclerosis (MS) is an autoimmune disorder commonly characterized by demyelination of neuronal axons and subsequent neurodegeneration [132,133]. Symptoms are widely varied, ranging from vision issues, muscle stiffness, and dizziness to mental/physical fatigue, mood swings, and cognitive defects [134]. The closest genetic links found for MS is the chromosome 6p21 major histocompatibility complex (MHC), which is a group of genes functioning in the immune system, including the human leukocyte antigen (HLA) gene cluster [135]. Environmental factors such as Vitamin D deficiency, smoking, obesity, and infections with the Epstein–Barr virus have been linked to MS onset [136,137,138]. Chronic inflammation is a frequent component of MS pathology and often results in oxidative stress [139]. Indeed, MS patients’ CNS tissue and peripheral mononuclear blood cells (PMBCs) show elevated 8-OH-dG, a marker of oxidative damage [133,140]. In addition, stained MS patient neurons show increased accumulation of oxidized phospholipids, an indication of oxidative damage, as well as degenerating dendrites [139]. Although DSBs in MS neurons remain largely unexplored in MS cases, inflammation-mediated oxidative DNA damage causes astrocytes and glia degeneration, leading to reduction in neuronal support and neurodegeneration in MS pathology.

### 3.5. Huntington’s Disease

Huntington’s disease is defined by a trinucleotide expansion of the CAG motif in the *HTT* gene, with pathology associated with of 40 or more repeats [141]. The expansion results in expression of a polyglutamine chain poly-Q in the Htt protein, causing it to aggregate and disrupt various cellular processes, leading to neuronal death. Clinical manifestations range from muscular issues to cognitive defects [142]. Human and mouse models alike display increased DNA damage, often preceding neuronal death [143,144,145,146]. For instance, ectopic expression of mutant Htt in mouse primary neurons resulted in increased DSBs [147]. Moreover, other studies show mutant Htt actively promoting defective DNA repair [6]. Mutant Htt directly interacts with various DNA repair proteins in the NHEJ pathway, during oxidative damage repair and transcription-coupled repair (TCR), and thus exacerbates DNA damage-based neurodegeneration [6,143,147]. Additionally, the DNA repair glycosylase OGG1 expands the CAG motif by repairing lesions in the area, contributing to disease development over time [148]. Lastly, DNA repair proteins, such as MLH1 and MSH2, often contribute to the trinucleotide expansion in Htt as a result of slippage during replication, highlighting a role for DNA damage as an initiator and contributor in HD pathology [149,150,151].

## 4. Histone PTMs Associated with DNA Double-Strand Breaks

**Figure 2:**
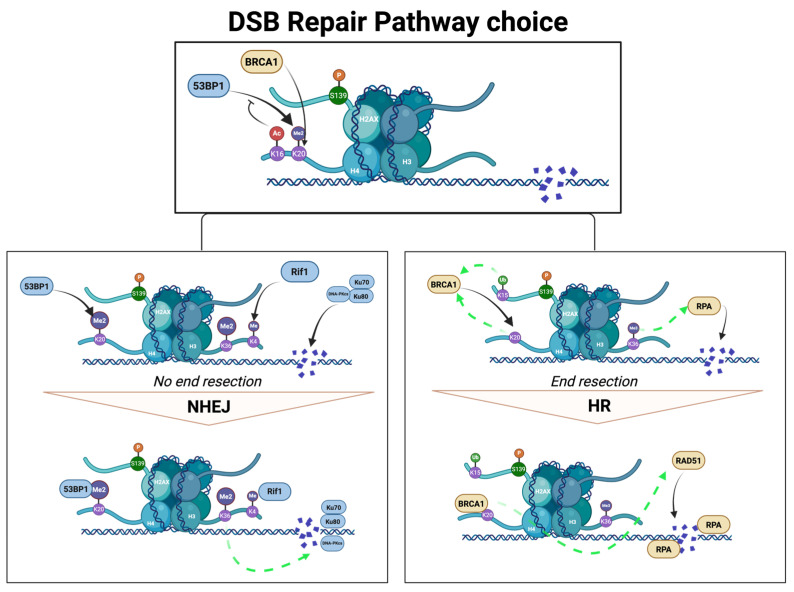
**Histone PTMs involved in DNA double-strand break (DSB) repair.** Interplay between p53-binding protein (53BP1) and breast cancer 1 protein (BRCA1) dictate pathway choice in DNA DSB repair. The binding partner histone PTM of these proteins and neighboring PTMs influence the recruitment of these repair proteins at the DSB sites, thereby also dictating repair pathway. Solid black arrows indicate recruitment, inhibitor arrows indicate inhibition, and dashed green arrows represent a stabilizing/supporting effect. Figure created in BioRender.

The post-translational modification of histones has been linked to DNA damage repair both as a signal to initiate as well as serving as binding platforms for repair proteins. Histone PTMs play important roles in DNA repair pathway choice and coordinate the molecular arrangement of repair proteins conducive to successful repair (Table 1 & Figure 2). In other words, histone PTMs enable the decision between NHEJ and HR and initiate DSB repair.

First, DSB sites accumulate Ataxia-Telangiectasia-Mutated (ATM), Ataxia-Telangiectasia and Rad3-Related (ATR), and DNA-dependent protein kinase catalytic subunit (DNA-PKcs) complexes which phosphorylate the histone variant H2AX at Serine 139 (known as the canonical DSB mark γ-H2AX) up to 1.7 Mb around the break site. Then, γ-H2AX coordinates with Ring Finger Protein 168 (RNF168) to recruit p53-binding protein 1 (53BP1) to promote NHEJ [60,61]. On the other hand, accumulation of Breast Cancer 1 (BRCA1) protein plays the determinant role in promoting HR [45,152,153]. Finally, the dynamic between 53BP1 and BRCA1 decides and coordinates the corresponding repair pathway [61,152]. Additional histone PTMs canonically known to be involved in DDR include H4K20me2, H2B-K120ub, and H3K36me3, among others, discussed below.

### 4.1. Histone PTMs in DNA Repair Pathway Choice

Histone PTMs contribute to the dynamic coordination between 53BP1 and BRCA1 in DSB repair pathway choice. While 53BP1 binds to various methylated states of H4K20, BRCA1 is linked to the unmodified H4K20 (H4K20unmod—also known as H4K20me0) often in crosstalk with H2AK15ub [154,155,156,157,158]. The presence of other certain neighboring PTMs can also affect the binding of these repair proteins, for example, they are implicated in recruitment and activation of ATM, as well as in γ-H2AX-based DSB foci formation in response to DNA damage. More importantly, H4K16ac sterically hinders the binding of 53BP1 and prevents NHEJ. Hence, H4K16ac/H4K20unmod engages HR [159]. On the other hand, dimethylation of H3K79 is implicated in recruiting 53BP1 to break sites [160,161]. Thus, H3K79me2/H4K20me2 engages NHEJ.

### 4.2. Histone PTMs and Homologous Recombination

Once HR is selected as the DSB repair pathway, histone PTMs directly enable it and facilitate end resection to promote faithful repair. End resection is a cleavage process that generates single-strand overhangs to prepare the DNA for HR [62]. During replication, the constitutive H4 methylation is diluted, and HR is promoted, indicating cell-cycle-dependent repair pathway choice. BRCA1 coordinates with the MRN complex, CtIP, EXO1, and BLM helicase via pre-existing H3K36me3 to resect DNA and generate single-stranded 3′ DNA overhangs [162]. Additionally, H3K36me3 promotes quick accumulation of Replication Protein A (RPA) at the break site, which then coats the overhang to prevent ssDNA folding and digestion [26,163]. Subsequently, RAD51 and BRCA2 accumulate at the DSB site and work together to facilitate strand invasion with a sister chromatid to promote faithful DNA synthesis for the damaged strand [164,165]. Other histone PTMs implicated in promoting HR include H3K9me3 and H3K4me2/3, which are traditionally known to enable transcription repression in DNA repair factor accumulation and BRCA1 recruitment [26]. Lastly, H4 acetylation on lysine 12 and lysine 16 are generally linked to promoting sister chromatid-mediated repair in HR [166].

**Table 1:** **Histone post-translational modifications (PTMs) associated with DNA double-strand break repair.**

**Repair Pathway**	Associated Histone PTMs	Repair Protein Partners	References
Homologous recombination	H2AK15ub H3K4me3 H3K9me3 H3K36me3 H3K79me3 H4K12/16ac H4K20meunmod	BLM BRCA1 BRCA2 CtIP EXO1 RAD51 RPA	[26] [162] [165] [167] [168] [169] [42]
Non-homologous end joining	H1ub H3K4me H3K36me2 H3K79me2 H4K16ac (debated)	53BP1 DNA-PKcs Ku70/Ku80 LIG4 XRCC4	[158] [166] [170] [171] [172]
Both	H2AXS139ph H2BK20ub H2BK123ub H3S10ph	MRN complex	[165] [167] [173] [174] [175]

### 4.3. Histone PTMs and Non-Homologous End Joining

Although a shorter repair cascade, NHEJ also involves various histone PTM-mediated steps. The NHEJ pathway initiates with the Ku70/Ku80 heterodimer binding at the DSB site, followed by autophosphorylation of DNA-PKcs. Interestingly, histone methylation tends to display context- or residue-specific effects, as well as different effects, depending on the methylation count. While H3K36me3 promotes HR, H3K36me2 enhances NHEJ [170,176]. H3K36me2 stabilizes the binding of Ku70 at DSB sites for subsequent repair [176]. Moreover, H3K4me negatively regulates end resection to promote DSB repair via NHEJ as part of the 53BP1-RIF1-shieldin pathway [60,171]. Finally, although the presence of H4K16ac is argued to be important for HR, some have shown that it could be beneficial for NHEJ in yeast [177].

### 4.4. Histone PTMs Involved in Both Homologous Recombination and Non-Homologous End Joining

Primarily associated with the cell cycle and not as a canonical DDR mark, H3S10ph has been linked to DDR in various indirect ways [57]. H3S10ph levels are known to be dynamic during the cycles of mitosis [178]. This PTM is involved in chromatin condensation in *S. cerevisiae* by recruiting the histone deacetylate HST2 to deacetylate H4K16ac [179]. H3S10ph also aids in the nuclear organization of replicating genes and transcribing chromatin away from the lamina rich periphery, as well as preventing the spread of repressive chromatin by antagonizing H3K9me2 [180]. In response to ionizing radiation, H3S10ph and γH2AX display inverse levels during the G1 phase of the cell cycle in the process of chromatin condensation to aid in repair by decreasing transcription [168,181]. Furthermore, DNA damage leads to Aurora B kinase inhibition via PARP-1 activation, which results in loss of H3S10ph on compact DNA during mitosis [182]. Dysregulated levels of H3S10ph in these contexts indicate susceptibility to genomic instability and damage, as well as impaired repair [57]. Increased H3S10ph levels are implicated in the formation of R-loops and concurrent chromatin condensation, leading to DNA fragility and damage, indicating the potential of H3S10ph enabling damage to DNA [183]. Additionally, H3S10ph hampers DNA damage repair via NHEJ during the G1 phase of the cell cycle as inhibition of MKP1, which dephosphorylates H3S10ph upon irradiation, resulting in cell death [181,184]. On the other hand, H3S10ph mediates dissociation of heterochromatin protein 1 (HP1), which aids in chromatin compaction and gene silencing [185]. Thus, H3S10ph-mediated loss of HP1 from chromatin enables DNA accessibility and repair protein recruitment for HR, furthering its role in DNA damage repair and DNA disrepair [167]. On the other hand, unwanted increased H3S10ph levels can dissociate HP1, randomly exposing DNA to damage via R-loops. Although these reports allude to a potential role for H3S10ph in DNA damage and repair, additional proof supporting this premise is currently lacking. Further mechanistic investigations are necessary to clarify the degree to which H3S10ph might be involved in DNA repair and identify whether its role is antagonistic or supportive.

Various other histone PTMs have been linked to both HR and NHEJ, highlighting a robust therapeutic avenue in treating neurodegeneration. H2AXS139ph, commonly known as γH2AX, is thoroughly implicated in both pathways. In addition to acting as a marker of DSBs and initiating repair, γH2AX engages in crosstalk with H3K79me3, H3S10ph, and H4K16ac in facilitating DSB repair [168,177,181,186]. Lastly, ubiquitination on H2A and H2B residues is linked to general repair cascade as well. Specifically, H2BK20ub and H2BK123ub have been implicated in both HR and NHEJ via DNA damage checkpoint signaling. Ubiquitination on H1, such as H1K17ub2, is linked to RNF168 activation, which then recruits 53BP1 and promotes H2AK12/15ub as part of DSB repair [169,173,174,175]. On the other hand, H1.2ub represses RNF168 recruitment and 53BP1 foci formation, thereby deterring NHEJ [187]. Other DDR relevant PTMs include H4S1ph, H4K20me, and H3K14/K23ac (influenced by H3S10ph) [188]. H3K56ac is debated; some claim it is required for chromatin reorganization after repairing DSBs arising from replication [189,190]. Evidently, further investigations into the dysregulation of histone PTMs can aid in finding new targets to attenuate DNA damage-based neurodegeneration.

### 4.5. Histone Variants Involved in DNA Double-Strand Break Repair

Several histone variants have been linked to DNA damage and repair. In addition to H2AX, H2AZ is another variant of H2A linked to DSB repair. While early deposition of H2AZ aids in NHEJ, subsequent removal of H2AZ enables HR [43,191]. Acetylation of various residues on H2AZ colocalize with γH2AX and contributes to DNA repair [192,193]. Moreover, macroH2A, specifically mH2A1, is another H2A variant that has been implicated in DSB repair [194,195]. It aids in DSB repair pathway choice in conjunction with PR domain zinc finger protein 2 (PRDM2). Subsequently, mH2A1 participates in HR via BRCA1-dependent processes [196]. Furthermore, CENPA is a H3 variant primarily localized in centromeres. Relocation of CENPA from centromeric regions to DSBs is also observed in response to DNA damage [197,198]. In addition to being recruited at DSBs, CENPA has been implicated in HR via its interaction with its chaperone Holliday Junction Recognition Protein (HJURP) [199]. Lastly, H3.3, the most homologous variant of canonical H3, is also involved in DDR. Interestingly, this variant has been linked to both NHEJ and HR [200,201]. Further investigation into the roles of histone variants and their modified versions will expand mechanistic investigations into epigenetic cellular process in diseases, potentially aiding in revealing novel targets for treatment.

## 5. Double-Strand Break Repair in Neurodegenerative Diseases—An Epigenetic Perspective

**Figure 3:**
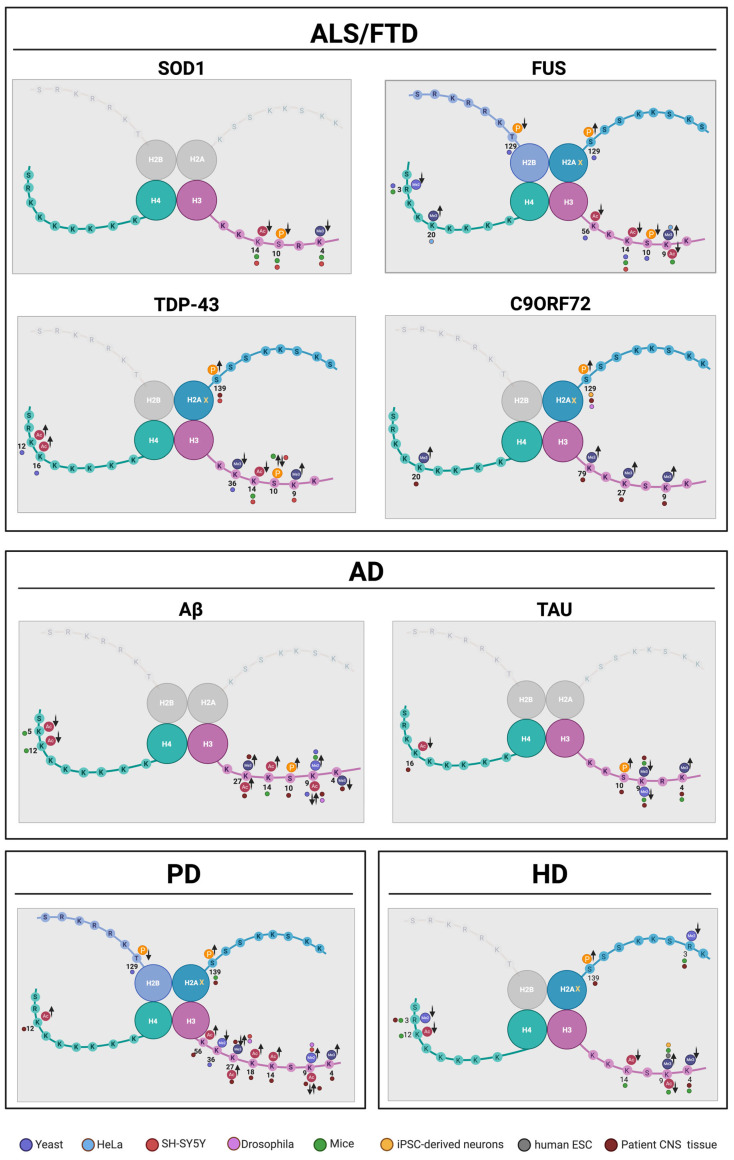
**Histone post-translational modifications (PTMs) associated with neurodegenerative diseases.** Various models display histone PTM dysregulations in ALS/FTD, AD, PD, and HD. Up arrows indicate increased levels while down arrows indicate decreased of PTMs. Ac = acetylation, Me = methylation, Me2 = dimethylation, Me3 = trimethylation, and P = phosphorylation. Model legend indicated. For references and description of models, refer to Table 2. Figure created in BioRender.

Histone PTMs play an important role in DNA damage response and repair. DNA double-strand breaks are the most severe type of damage, arising from unrepaired single-strand breaks, radiation, and chemotherapeutics. DSBs are repaired via either homologous recombination or non-homologous end joining. As neurons are post-mitotic, it is essential that DSB repair occurs successfully for proper CNS function. Based on current evidence, it is clear that DNA damage and defective repair contribute to the etiology of various neurodegenerative diseases. Therefore, it is possible that dysregulation of histone PTMs associated with DDR, specifically DSB repair, may provide further insight into the mechanistic role of DNA damage in neurodegeneration and illuminate novel epigenetic targets to tackle neurodegeneration via manipulation of DDR. Below, we discuss histone PTM alterations associated with DSBs observed in NDs in various eukaryotic models ranging from yeast to patient-derived cells (Figure 3).

The models discussed here range from the unicellular eukaryote *Saccharomyces cerevisiae* to synthetic cellular models with induced disease phenotypes and disease models directly derived from patients such as fibroblasts and induced pluripotent stem cells (iPSCs). Firstly, while yeast are single-cell organisms that do not embody a nervous system and lack neuron-specific structures, the high homology between yeast and human cells enable their use as an easily manipulable cellular model of disease. Humanized yeast can aid in discerning specific effects of genes and aberrant protein functions, as well as pathway analyses homologous with human cells. On the other hand, synthetic models such as SH-SY5Y and HeLa cells expressing disease-associated toxic proteins generally do well to model disease phenotypes and aid in target validation as well as in testing potential therapeutics. However, it is important to note that these cells display cancer-related phenotypes and toxicities, such as dysregulated cell cycle checkpoints, altered DNA repair pathways, and genetic instability.

### 5.1. Amyotrophic Lateral Sclerosis/Frontotemporal Dementia

#### 5.1.1. SOD1

ROS-mediated DNA damage and ineffective repair pathways are connected to SOD1 mutations and the resulting defects in the antioxidant properties of SOD1 [202]. SH-SY5Y and transgenic mouse models of ALS-SOD1 mutations show decreased levels of H3K4me2, H3S10ph, and H3K14ac [203]. H3K4me2 has been linked to HR due to its binding to the DNA end resection agent Rif1, while H3S10ph has been linked to DNA repair in nuclear organization and initiating DNA repair induced by γ-H2AX in both the G1 and G2 phases [168,180,181]. Additionally, H3K14ac stabilizes the RSC (remodeling the structure of chromatin) complex to remodel the chromatin, aiding repair in response to UV damage [188]. Decreased levels of these PTMs could imply that in addition to reduced ROS removal, mutant SOD1 neurons experience decreased DNA repair engagement and efficiency.

#### 5.1.2. FUS

In addition to mislocalizing to the cytoplasm and aggregating, mutant FUS sequesters proteins with essential functions in energy metabolism, DNA repair, protein synthesis, as well as RNA metabolism [82,204,205]. FUS-ALS models reveal histone PTM alterations. Notably, yeast cells overexpressing FUS show increased H2AS129ph levels, the yeast equivalent of γ-H2AX [206,207]. Our own work in yeast overexpressing FUS identified decreased levels of H2B129ph, H3S10ph, H3K14ac, H3K56ac, and H4R3me2 [208]. While decreased levels of H3S10ph could indicate DNA repair signaling, decreased levels of H3K56ac indicate potential loss of post-repair cleanup.

In mammalian models, decreased levels of H3K9ac and H3K14ac in mouse spinal cord cultures expressing mutant FUS (R521H) were tied to FUS’s mislocalization to the cytoplasm [209]. Furthermore, HeLa cells overexpressing FUS show increased levels of H3K9me3, a widely known active chromatin mark, as well as increased levels of H4K20me3 at telomeres [210]. Both of these allude to the HR pathway, with H3K9me3 aiding in increased chromatin accessibility while H4K20me3 promotes 53BP1 binding at damage sites when H4K16ac is absent. Additionally, SH-SY5Y cells virally treated with a FUS mutant protein (R495X) display decreased H3S10ph and H3K14ac levels, further indicating dysregulation of repair as these marks are involved in preparing DNA for repair [203]. Moreover, mouse neurons expressing human FUS show decreased levels of asymmetric H4R3me2 and H3K9ac [209]. Asymmetric H4R3me2 has been linked to transcriptional activation and by extension chromatin accessibility, while elevated H3K9ac has context-dependent function in DNA repair. On the other hand, H3K14ac is linked to recruitment of SW1/SNF chromatin remodeling complex to γ-H2AX and activation of ATM to facilitate DSB repair [42]. Decreased levels of H3S10ph, H3K14ac, and H4R3me2 in both yeast and mammalian models suggest that decreased chromatin accessibility and improper machinery positioning contribute to defective repair and potential neurodegeneration.

#### 5.1.3. TDP-43

TDP-43 aggregation disrupts RNA transport processes via formation of stress granules [89]. Additionally, TDP-43 has been widely implicated in ALS-related neurodegeneration in association with DNA damage [7,92]. TDP-43 pathology has been linked to quite a number of histone PTMs. In agreement with increased DNA damage in ALS, SH-SY5Y cells expressing a Q331K mutation on TDP-43 and patient spinal cord tissue show increased H2A/XS139ph levels, indicating potential repair initiation [93]. However, neuronal death suggests potential repair dysregulation or aberrant signaling causing neurodegeneration. Fitting in with this hypothesis, SH-SY5Y cells transduced with mutant TDP-43 (M337V) reveal decreased levels of H3S10ph and H3K14ac, while SH-SY5Y cells overexpressing wild-type TDP-43 display increased H3K9me3 levels compared to cells with basal level TDP-43 expression [203].

In yeast overexpressing TDP-43, H4K16ac, a histone mark known to induce loosening of nucleosomes and promote BRCA1 accumulation in HR, is increased [208]. However, the same yeast model cells show decreased levels of H3K36me3, which is involved in facilitating HR, indicating dysregulated DNA repair signaling. Other altered PTMs include increased levels of H4K12ac, which is involved in RAD51 recruitment for HR [201,208]. Taken together, H3K36me3’s role in promoting HR and H4K16ac and H4K12ac participating in DNA damage signaling and repair suggest that DDR is engaged to some signaling degree, but potential misalignment downstream may be preventing successful DNA repair and leading to neurodegeneration.

#### 5.1.4. CORFf72

C9ORF72 has roles in autophagy, nucleocytoplasmic transport, and DNA repair [63,99,103]. The HRE expansion in *C9orf72*, which results in loss-of-function of C9ORF72 and gain-of-function of RNA and DPR aggregates, affects these processes. C9-ALS patient motor neurons, iPSC-derived neurons with ectopic DPR expression, and fly neurons expressing DPRs display elevated H2A/XS139ph and p53 activation [100,104,211]. Moreover, various models, including neurons from rats, human cells, and C9 patient spinal cord tissues, display increased γ-H2AX levels and perturbed ATM signaling, a crucial step for the repair cascade, indicating increased DNA damage and defective repair [212]. Furthermore, increased levels of H3K9me3, H3K27me3, H3K79me3, and H4K20me3 occur in C9 patient frontal cortex and cerebellum [213]. While H3K9me3 has been linked to reduction of a repressive state of chromatin to aid in repair, H3K27me3 induces a repressive state which negatively affects repair pathways by potentially limiting DNA accessibility. H4K16ac and H4K20me3 work on mutually exclusive repair pathways for DSB repair [155,159].

Although yeast lack a nervous system, they provide insight into the isolated and direct effect of mutations on specific phenotypes in a simpler cellular model. As such, our own work in yeast overexpressing DPR Proline-Arginine (poly-PR) has found alterations in a number of histone PTMs [214]. Curiously, despite the absence of cell cycle disturbances, we find a genome-wide increase in H3S10ph levels. Furthermore, we also detect increases in H3K36me3, H3K79me3, and H4K16ac, all of which have been associated with DNA damage response signaling, specifically in the context of HR [156,163,215]. Clearly, DNA repair-related signaling could be leading to persistent DNA damage and contributing to cell death and neurodegeneration. However, further investigation is required to definitively establish these associations.

### 5.2. Parkinson’s Disease

α-Syn aggregates form Lewy body inclusions in neurons that propagate to other cell types and brain regions in a prion propagation-like manner [216]. Various histone PTM marks have been found to be altered in PD models. Interestingly, an α-Syn yeast overexpression model displayed decreased levels of H2BT129ph and H3K36me2, marks linked to DNA damage and repair potentially indicating ineffective repair [208]. Neuronal samples of transgenic Drosophila display increased H3K9me2 and H3K27me3 levels [217]. In mammalian models, SH-SY5Y expressing α-Syn agree with these observations in H3K9me2 and H3K27me3 [217]. Furthermore, a PD mouse model established from viral-delivered α-Syn displays increased DSB foci formation, marked by γ-H2AX and 53BP1 accumulation [107]. Similarly, two different in vivo mouse PD models display increased γ-H2AX levels [107,113].

Patient tissues also display histone PTM changes related to DNA damage. For instance, PD patients’ brains display increased γ-H2AX levels compared to age-matched control patients [107,218,219]. Moreover, PD patients’ post-mortem motor cortices display decreased levels of H3K9ac and increased levels of H3K14ac and H3K18ac [220]. According to another report, substantia nigra neurons from PD patient brains also display increased H3K4me3 and H3K27ac and decreased levels of H3K27me3, indicating promotion of DNA repair, contradicting observations in Drosophila and SH-SY5Y [221]. Lastly, a pan-histone acetylation analysis of the prefrontal cortex of PD patients revealed increased levels of H3K9ac, H3K14ac, H3K27ac, H3K56ac, and H4K12ac [222]. The same study shows that while the cerebellum corroborated the elevated H3K9ac and H3K14ac levels, the striatum corroborated the increased levels of H3K27ac [222]. As mentioned before, H3K56ac has been associated with post-repair cleanup, and H3K9ac has been linked to DNA repair processes, as well as inducing transcription of repair genes. H4K14ac recruits the chromatin remodeler Rsc family to facilitate repair [188]. While not strictly related to DNA repair, H3K27ac can be turning on genes that aid in repair, and acetylation indicates general chromatin accessibility. All in all, α-Syn PD histone PTM disturbances suggest DNA damage repair signaling, as widespread histone acetylation suggests DNA preparation for DSB repair [42]. Further work is necessary to determine whether a repair pathway choice conundrum or repair mechanism imbalance leads to persistent neuronal damage.

### 5.3. Alzheimer’s Disease

#### 5.3.1. Amyloid-β

Aβ-AD pathology involves accumulation of Aβ plaques that disrupt membrane-associated cellular processes. Certain histone PTMs are linked to Aβ-AD pathology. For instance, yeast overexpressing Aβ 1–40 display decreased H3K9ac and increased H3K9me2 levels [223]. Furthermore, mice with APP Aβ-pathology show decreased levels of H4K5ac and H4K12ac [224]. H4 acetylation is generally involved in chromatin structure changes and increased chromatin accessibility. Notably, H4 acetylation facilitates HR by promoting MDC1, BRCA1, and RAD51 accumulation at DNA DSB sites [42,177,225]. Mice overexpressing mutAPP also show increased H3K14ac and H3K9me2 [226]. Moreover, AD patient neurons show increased levels of H3S10ph, a cell cycle linked histone PTM. Interestingly, neuronal cell cycle re-entry has been implicated in AD [227]. As cells marked for cell cycle re-entry ultimately die, this begs the question of why the cells re-enter cell cycle in degenerative contexts. Furthermore, decreased levels of BRCA1 in AD cases, despite histone hyperacetylation seen in Aβ cases, would suggest aberrant DDR. The resulting persistent damage could be contributing to neuronal death, as cell cycle reentry often leads to apoptosis [228,229]. Interestingly, a multi-omics wide-scale histone PTM scan of AD post-mortem human brains show enrichment of H3K27ac and H3K9ac [230]. This is also observed in a fly model expressing Aβ suggesting a feedback loop between the Aβ-derived neurodegeneration coupled with the histone PTM-based exacerbation [230]. Furthermore, AD patient brain cortices display decreased H3K4me3 levels and increased H3K27me3 levels [231]. Although H3K4me3 and H3K27me3 are more commonly known for transcriptional regulation at gene promoters, some have shown that loss of H3K4me3 occurs at damage sites to aid in damage signaling for repair, while H3K27me3 may inhibit repair by repressing chromatin [232,233,234]. Coexistence of histone marks involved in repression and de-repression of chromatin suggest genomic instability and aberrant DDR.

#### 5.3.2. TAU

TAU also has been linked to DDR in AD. TAU is thought to directly interact with chromatin and cause changes setting up a feedback loop furthering genomic damage. In a genome-wide profile study, H4K16ac levels were severely decreased in AD patient brains as shown [235]. A TAU-AD mouse model, a transgenic TAU-AD fly model, and human AD brains display decreased H3K9me2 levels as well [131]. Another report, however, shows increased H3K9me2 levels in a mouse AD model, as well as patient prefrontal cortices [226]. According to another report, AD human prefrontal cortices and TAU transgenic mice show increased H3K4me3 levels [236]. As H3K4me3 and H3K9me2/3 are involved in promoting and facilitating HR, different levels of each indicate abnormal damage signaling and repair [26]. Additionally, TAU-AD patient brains indicate increased levels of H3S10ph, agreeing with the notion of cell cycle reentry in AD along with mitotic machinery activation, although not necessarily linked to TAU [229,237,238,239]. H4K16ac and H3S10ph participate in crosstalk in transcriptional elongation and chromatin structure dynamics and are, therefore, indirectly involved in DNA repair. Since H3S10ph levels are meant to decrease in response to DNA damage to facilitate repair, the increased levels in AD point to repair dysregulation [57]. Although H3K4me3 is a mark of active gene expression, it can also be indicative of transcription-coupled repair. Current evidence points to atypical DDR and TAU-AD pathology synergizing to influence cell cycle re-entry. Further focused work is necessary to gain a better understanding of the histone PTM dysregulation and their link to DDR-associated neurodegeneration in TAU-AD.

### 5.4. Multiple Sclerosis

Multiple sclerosis is characterized by the demyelination of neurons or the degradation of oligodendrocytes that myelinate neuronal axons. As such, loss of myelination affects neuronal conductivity and overall brain function. Although epigenetic investigations in MS neurodegeneration are thus far lacking, some tissue-based experiments reveal HME and histone PTM involvement. For example, examination of MS brains shows increased H3 acetylation levels compared to controls, along with increased levels of corresponding histone acetyltransferases (HATs) [240]. Fitting in with this finding, a mouse model (autoimmune encephalomyelitis) with reduced HDAC activity shows MS symptom improvement [241]. As MS pathology is studied predominantly in oligodendrocytes, more research in neurons is required to understand whether and how the neurons tackle the loss of myelination in the epigenetic front, specifically via histone PTMs.

### 5.5. Huntington’s Disease

Huntington’s disease results from a CAG repeat nucleotide expansion giving rise to polyglutamine (polyQ) peptides [242]. Although investigations are ongoing, various models of HD indicate a role for histone PTMs in disease pathology, as well as DNA damage, as demonstrated by increased γ-H2AX levels in HD patients’ striata [147]. Furthermore, transgenic mouse models display locus-specific decreased levels of H3K9ac and H3K14ac in the hippocampus and cerebellum. Decreased H4K12ac levels were also observed in this model supporting deacetylation in HD pathology [243]. As acetylation aids in increasing chromatin accessibility, which is necessary for repair, the decreased acetylation levels in these PTMs could indicate defective repair. Additionally, a transgenic mouse model displays H3K9 and H3K14 deacetylation in the striatum [244]. Furthermore, primary mouse cortical neurons transfected with full-length mutant Htt show decreased levels of R3Me2 on both H2A and H4 [245].

Data from HD patients also supports a role for DNA damage and histone PTMs in this disease. For instance, ChIP-seq analysis of prefrontal cortex neuron human HD cases show decreased H3K4me3 [173,241]. Another investigation in 12 wk HD mousestriata and cortices display increased levels of H3K4me3 in downregulated genes in correlation with CAG expansion [246]. Moreover, human embryonic cells (hESCs) expressing mutant Htt via lentiviral infection and a transgenic HD mouse model display increased H3K9me3 levels [247,248]. Concurrent decreases in H3K4me3 levels and increases in H3K9me3 levels make a case for tightly packed DNA inaccessible for repair. Reduction in H2A/H4R3me2 levels is also found in front superior cortices from HD patients [245]. H4R3me2 promotes chromatin accessibility for gene transcription as well as oxidative damage repair. Hence, a reduction in this PTM in HD suggests defective repair [249,250]. The available evidence highlights that defective DDR has a role in HD pathology, and further investigation could highlight key targetable players.

A summary of all histone PTM changes in the neurodegenerative diseases reviewed is presented in Table 2, as well as in Figure 3.

**Table 2:** **Histone post-translational modifications (PTMs) associated with ALS/FTD, PD, AD, MS, and HD.** Orange color indicates increased PTM levels while blue indicates decreased PTM levels.

Disease	Mutation	Model	Dysregulated Histone PTMs	References
ALS/FTD	SOD1	SH-SY5Y (G93A and H80R)	H3K4me2 H3S10ph H3K14ac	[203]
		Mice (G93A)	H3K4me2 H3S10ph H3K14ac	[203]
	FUS	Yeast (FUS overexpression)	H2AS129ph	[206,207]
			H2B129ph H3S10ph H3K14ac H3K56ac H4R3me2	[208]
		Mice (R521H)	H3K9ac H3K14ac	[209]
		Mice (human FUS)	H3K9ac H4R3me2	[209]
		HeLa (FUS overexpression)	H3K9me3 H4K20me3	[210]
		SH-SY5Y (R495X)	H3S10ph H3K14ac	[203]
	TDP-43	Yeast (TDP-43 overexpression)	H3K36me3 H4K12ac H4K16ac	[201,208]
		SH-SY5Y (Q331K)	H2A/XS139ph	[93]
		SH-SY5Y (M337V)	H3S10ph H3K14ac	[203]
		SH-SY5Y (TDP-43 overexpression)	H3K9me3	[203]
		Patient CNS tissue	H2A/XS139ph	[93]
	C9ORF72	Yeast (poly-PR)	H3S10ph H3K36me3 H3K79me3 H4K16ac	[214]
		C9-ALS motor neurons	H2A/XS139ph	[104]
		iPSC-derived neurons (ectopic DPR expression)	H2A/XS139ph	[100,104]
		Drosophila (DPR expression)	H2A/XS139ph	[92,101,220]
		Patient CNS tissue	γ-H2AX	[212]
			H3K9me3 H3K27me3 H3K79me3 H4K20me3	[213]
PD	-	Yeast (α-Syn overexpression)	H2BT129ph H3K36me2	[208]
		Drosophila (α-Syn overexpression)	H3K9me2 H3K27me3	[217]
		SH-SY5Y (α-Syn overexpression)	H3K9me2 H3K27me3	[217]
		Mice (injected α-Syn)	γ-H2AX	[107]
		Mice (viral-delivered α-Syn (A53T))	γ-H2AX	[113]
		Patient CNS tissue	γ-H2AX	[107,218,219]
			γ-H2AX H3K9ac H3K14ac H3K18ac	[220]
			H3K4me3 H3K27ac H3K27me3	[221]
			H3K9ac H3K14ac H3K27ac H3K56ac H4K12ac	[222]
AD	Amyloid-β	Yeast (Aβ 1–40 overexpression)	H3K9ac H3K9me2	[223]
		Drosophila (Aβ overexpression)	H3K9ac H3K27ac	[230]
		Mice (APP-Aβ overexpression)	H4K5ac H4K12ac	[224]
		Mice (mutAPP overexpression)	H3K9me2 H3K14ac	[226]
		Patient neurons	H3S10ph	[225]
		Patient CNS tissue	H3K9ac H3K27ac	[230]
			H3K4me3 H3K27me3	[231]
	TAU	Mice (TAU overexpression)	H3K4me3	[236]
			H3K9me2	[131]
		Patient CNS tissue	H4K16ac	[235]
			H3K9me2	[131]
			H3K4me3	[236]
			H3S10ph	[237]
MS	-	Patient CNS tissue	H3-ac	[240]
HD	-	Mice (transgenic)	H3K9ac H3K14ac H4K12ac	[243]
			H3K9ac H3K14ac	[244]
			H3K9me3	[248]
		hESC (viral mutHtt)	H3K9me3	[247]
		iPSC (viral mutHtt)	H3K9me3	[247]
		Mice (transfected mutHtt)	H2AR3me2 H4R3me2	[247]
		Patient CNS tissue	γ-H2AX	[146]
			H3K4me3	[172,251]
			H2AR3me2 H4R3me2	[245]

## 6. Conclusions

Neurodegenerative diseases arise and progress via numerous overlapping mechanisms. Protein aggregation, defective RNA metabolism, ER stress, and DNA damage are all active contributors to these pathologies. DNA damage, particularly, has a significant role in promoting neuronal damage via the damage itself, as well as defective repair. DDR, therefore, deserves strong attention in further understanding neurodegeneration onset and progression. Neurons are terminally differentiated and accumulate much damage from carrying out metabolic functions over their life span. Specifically in cases of DNA double-strand breaks, persistent DSBs cause cells to resort to apoptosis. In addition, if repair mechanisms are defective, the damage coupled with the hyperactive DDR signaling could overwhelm the repair system and harm the cells instead of promoting repair. Moreover, persistent DNA damage can cause cell cycle re-entry, which is an abortive and fatal process. As cell cycle re-entry is usually a direct effect of the loss of suppression of cell cycle genes in neurons, further investigation is necessary to determine the connections between DNA damage and cell cycle gene activation in these contexts. As such, genes and proteins at the conjunction of DNA repair pathways, cell cycle re-entry, and neuronal death require closer examination. Thus, the study of DDRs in neurodegeneration may illuminate targetable proteins and pathways to enhance repair or halt aberrant repair processes contributing to neuronal death.

## 7. Future Directions

Epigenetics-based therapeutics, or epidrugs, hold great promise against neurodegenerative diseases. Current treatments against these ailments target symptoms rather than targeting the disease itself. For example, prominent FDA-approved treatments against ALS such as riluzole and edaravone, moderately improve quality of life for patients rather than halt disease progression. However, recent studies show that epidrugs such as HDAC inhibitors hold promise in ALS/FTD, AD, and PD [252,253,254,255]. Indeed, some have entered clinical trials with demonstrated promise [256,257]. The numerous epidrugs in preclinical trials against cancer can be investigated in NDs, as many of the hallmarks of cancer and NDs overlap [258]. Histone PTMs can also be used as blood biomarkers for diagnostic purposes in peripheral mononuclear blood cells via ChIP against disease-associated PTMs [213]. Novel treatment techniques such as CRISPR-mediated gene editing would be better informed by understanding DNA repair pathways, specifically in post-mitotic cells like neurons [259]. Further investigating histone PTMs related to DDR can expand the target search to better facilitate repair or inhibit aberrant repair to tackle neuronal degeneration.

DDR-associated histone PTMs can become valuable targets against neurodegeneration etiologies. Histone PTMs serve as scaffolds and work closely with DNA repair proteins to facilitate repair. In the various stages and pathways of repair, disrepair and dysregulation can be targeted. Furthermore, histone PTMs are highly dynamic and pharmacologically accessible. The recent literature shows that overactive and dysregulated DDR can be targeted to tackle neurodegenerative pathologies. Various models of CNS neurodegeneration, including optic nerve injury and spinal cord injury, as well as a Drosophila AD model with Aβ expression and a Drosophila HD model, show the neuroprotective effect of targeting DNA repair proteins, making a case for targeting DDR to relieve neurodegeneration [260]. Furthermore, a mouse PD model treated with pharmacological intervention against ATM, a key repair protein responsible for phosphorylating other repair proteins and initiating repair, shows decreased DSB levels and senescence-inducing proteins that further neurodegeneration [113]. As we reviewed, key histone PTMs involved in DDR include H3K36me3, H4K16ac, H3K79me3, and H4K20unmod in homologous recombination and H3K36me2, H4K20me2, and H3K4me in non-homologous end joining. Although currently unexploited, the plethora of histone PTMs involved in DNA repair and their respective HMEs, such as methyltransferases, acetyltransferases, (writers) and demethylases, deacetylases (erasers), as well as bromodomains and chromodomains (readers), present great potential for neurodegenerative disease treatment.

## Data Availability

No new data were created or analyzed in this study. Data sharing is not applicable to this article.

## Abbreviations

53BP1	p53 binding protein 1
8OH2′dG	8-hydroxy-2′deoxyguanosine
AD	Alzheimer’s disease
ALS	Amyotrophic lateral sclerosis
APP	Amyloid precursor protein
ATM	Ataxia-telangiectasia mutated
ATR	Ataxia-telangiectasia and Rad3-related protein
Aβ	Amyloid beta
BLM	Bloom syndrome helicase
BRCA1	Breast cancer gene 1
BRCA2	Breast cancer gene 2
BRD7	Bromodomain protein 7
C9ORF72	Chromosome 9 open reading frame 72
CAG	Cytosine–adenine–guanine
CENPA	Centromere protein A
CHD1	Chromodomain protein 1
CHK1	Checkpoint kinase 1
CNS	Central nervous system
CtIP	C-terminal binding protein-interacting protein
DDR	DNA damage response
DJ-1	Parkinsonism associated protein deglycase
DNA	Deoxyribonucleic Acid
DNA-PKcs	DNA-dependent protein kinase catalytic subunit
DPR	Dipeptide repeat protein
DSB	Double-strand break
ER	Endoplasmic reticulum
EXO1	Exonuclease 1
FTD	Frontotemporal dementia
FUS	Fused in sarcoma
hAPP	Human APP
HAT	Histone acetyltransferase
HD	Huntington’s disease
HDAC1	Histone deacetylase 1
hESC	Human embryonic stem cells
HLA	Human leukocyte antigen
HME	Histone-modifying enzyme
hnRNP	Heterogeneous nuclear ribonucleoprotein
HP1	Heterochromatin protein 1
HR	Homologous recombination
HST2	Homolog of Sir two 2
HTT	Huntingtin gene
iPSC	Induced pluripotent stem cell
KMT	Lysine methyltransferase
LIG4	Ligase 4 complex
MDC1	Mediator of DNA damage checkpoint 1
MHC	Major histocompatibility complex
MH2A	Macro histone H2A variant
MKP1	Mitogen-activated protein kinase phosphatase 1
MLL	Mixed lineage leukemia
MMR	Mismatch repair
MRE11	Meiotic recombination 11
MRN	MRE11-RAF50-MBS1 comples
MS	Multiple sclerosis
mutAPP	Mutant APP
NBS1	Nijmegen breakage syndrome 1
ND	Neurodegenerative Disease
NHEJ	Non-homologous end joining
NSC34	Neuroblastoma x Spinal cord hybrid 34 cell line
OGG1	8-oxoguanine DNA glycosylase 1
PARP	Poly(ADP-ribose) polymerase
PBMC	Peripheral blood mononuclear cell
PD	Parkinson’s disease
Poly-Q	Poly-glutamine
PR	Proline–arginine
PRDM2	PR domain zinc finger protein 2
PSEN1	Presenilin 1
PSEN2	Presenilin 2
PTM	Post-translational modification
RAD50	DNA repair protein RAD50
RAD51	RAD51 recombinase
RAN	Repeat-associated non-AUG
RGG	Arginine–glycine–glycine
RIF1	Replication timing regulatory factor 1
RNA	Ribonucleic acid
RNF168	Ring finger protein 168
ROS	Reactive oxygen species
RPA	Replication protein A
RSC	Remodeling the structure of chromatin complex
SAGA	Spt-Ada-Gcn5 acetyltransferase
SET1	Set1 protein product
sInDel	Somatic insertion/deletion
SNCA	Synuclein alpha gene
SOD1	Superoxide dismutase 1
ssDNA	Single-stranded DNA
sSNV	Somatic single nucleotide variant
TAU	Tubulin-associated unit
TCR	Transcription-coupled repair
TDP-43	Transactive response DNA-binding protein 43 kDA
UPR	Unfolded protein response
XRCC4	X-ray repair cross complementing 4
α-SYN	Alpha synuclein
